# Medical student self-efficacy, knowledge and communication in adolescent medicine

**DOI:** 10.5116/ijme.53d3.7b30

**Published:** 2014-08-20

**Authors:** Jennifer L. Woods, Tracie L. Pasold, Beatrice A. Boateng, Devon J. Hensel

**Affiliations:** 1 Division of Adolescent and Transition Medicine, Department of Pediatrics, Cincinnati Children’s Hospital Medical Center, USA; 2 Department of Psychology and Counseling, Marywood University in Scranton, USA; 3 Office of Education, Department of Pediatrics, University of Arkansas for Medical Sciences; 4 Division of Adolescent Medicine, Department of Pediatrics, Indiana University School of Medicine, USA

**Keywords:** Medical student, adolescent medicine, standardized patients, self-efficacy, medical student self-efficacy, knowledge and communication in adolescent medicine

## Abstract

**Objectives:**

To evaluate student self-efficacy, knowledge and communication with teen issues and learning activities.

**Methods:**

Data were collected during the 8-week pediatric rotation for third–year medical students at a local children’s hospital. Students completed a self-efficacy instrument at the beginning and end of the rotation; knowledge and communication skills were evaluated during standardized patient cases as part of the objective structured clinical examination. Self-efficacy, knowledge and communication frequencies were described with descriptive statistics; differences between groups were also evaluated utilizing two-sample t-tests.

**Results:**

Self-efficacy levels of both groups increased by the end of the pediatric rotation, but students in the two-lecture group displayed significantly higher self-efficacy in confidentiality with adolescents (t_(35)_=-2.543, p=0.02); interviewing adolescents, assessing risk, sexually transmitted infection risk and prevention counseling, contraception counseling were higher with marginal significance. No significant differences were found between groups for communication; assessing sexually transmitted infection risk was marginally significant for knowledge application during the clinical exam.

**Conclusions:**

Medical student self-efficacy appears to change over time with effects from different learning methods; this higher self-efficacy may increase future comfort and willingness to work with this high-risk, high-needs group throughout a medical career.

## Introduction

For third year medical students, the pediatrics rotation is typically a total of 6-8 weeks with a combination of inpatient and outpatient time. During this restricted timeline, students are exposed to subspecialty pediatrics, including adolescent medicine, for only a few days. These limitations are even more severe than with pediatric residents who have one month designated for adolescent medicine during the entirety of a three-year residency. With their one-month rotation, pediatric residents express their lack of knowledge regarding adolescent issues including sexuality, contraception, chronic illness, psychosocial issues and counseling.^1^^-^^3^ Medical students also emphasize discomfort with these difficult issues facing adolescents but recognize their need to learn skills such as interviewing to fully communicate with teens.^4^ Such limitations emphasize that as medical students learn basic medical information, it is important to establish a solid foundation with communication skills in order to raise awareness and sensitivity for working with patients early in medical training.^5^Over the years, medical students have learned from many teaching methods. Recently, medical students have shown higher satisfaction with case method learning compared to computer lectures and panel discussions as it facilitates interprofessional learning.^6^ In direct comparisons to traditional lectures, case learning had higher satisfaction scores.^7^ Additionally, health science students have described lecture-only learning as boring and a hindrance to understanding important topics.^8^Overall, the basic structure of instruction for medical students in respect to adolescent medicine is largely unknown. In fact, few studies have directly assessed medical students and adolescent medicine in any context. This is at least partially due to limited exposure during medical school. In studies with adolescents, students are often assessed at the end of rotations using standardized patients (SP) encounters or during their objective structured clinical examination (OSCE), an examination with clinical situations to test theoretical knowledge and clinical skills. The OSCE was created in 1975 but R. Harden and was developed to assess clinical competence by using multiple stations with varying clinical tasks such as history taking, physical examination, patient education, order writing, or test interpreting.^9^ In the current iteration, many OSCE exams involve standardized patients to allow a more realistic approach and immediate feedback regarding communication and observation of knowledge application. Kaul, Barley and Guiton (2012) showed in their study that medical students during their OSCE performed highly in communication and professionalism, but performed much lower in history-taking competence.^10^ Medical students have also assessed tobacco and alcohol with SPs. Overall, the history-taking skills of students and the satisfaction of SPs decrease as more complex patient risk factors are presented.^11^Another area that has not been explored for medical students and adolescent medicine is self-efficacy. According to Social Cognitive theory, the likelihood of an action occurring increases as belief in personal capability to execute an action also increases.^12^ Therefore, people undertake activities where they are likely to succeed, and self-efficacy is a reliable predictor of behavior.^13^ Previous work has examined the role of self-efficacy in medical student anatomy curriculums, family-centered care during bedside rounds, and overall success in medical school.^14^^-^^16^ Pre- and post-test evaluations are important components of many medical student self-efficacy studies as they measure the effects of the interventions.^17^^,^^18^ Another important facet of self-efficacy studies with students is often the use of standardized patients (SPs) to evaluate communication and knowledge skills.^19^^,^^20^Prior studies have evaluated medical student self-efficacy and medical skills in a general context. Despite these studies, there have been no assessments of medical student self-efficacy, knowledge usage or communication skill regarding adolescent medicine during the pediatric rotation. Specifically, the objectives of our study are (1) to evaluate the self-efficacy of third-year medical students at the beginning and end of their 8-week pediatrics rotation, (2) to assess the application of their knowledge base and communication skills in regard to adolescent health visits during their OSCE, and to (3) assess differences in self-efficacy, knowledge, and communication between medical students receiving a computer-based PowerPoint adolescent medicine lecture with narrative and medical students receiving the PowerPoint lecture plus an interactive case-based adolescent medicine learning activity.

## Methods

### Design

The study was initiated in March 2011 with third-year medical students during their pediatric clerkship. As part of the study, students completed an individual self-efficacy assessment during orientation. During their two-month rotation, the students rotated through the adolescent medicine clinic for one to three 4-hour sessions. To assist with their learning, students were also provided with online access to a 60 minute PowerPoint lecture with audio narrative presented by an adolescent faculty member regarding common diagnoses seen in adolescent medicine including growth and development, puberty, eating disorders, contraception, sexually transmitted infections (STI), sports medicine/injuries and gynecological issues. Two groups of students also participated in a 60-minute interactive case-based learning activity. The third group of students received only the PowerPoint lecture due to a change in curriculum secondary to faculty availability. Given this change in curriculum, the investigators modified part of the study for an assessment of differences between groups based on lecture(s) received.At the conclusion of the pediatric clerkship, students participated in an OSCE. The OSCE is a common examination tool used for medical student clerkships at the end of rotations in the United States medical school system. The OSCE acts as a test of clinical performance and competence in skills such as communication and examination. For this OSCE in pediatrics, four stations were completed by each student with a 10-minute time limit for each station. The stations were designed to test information learned during the rotation with application of the gained clinical and theoretical knowledge. Each station had a simulated patient portraying a child or a parent, and each case represented an issue commonly addressed in pediatrics. Students answered written questions at every station which counted as part of their final grade for the rotation, but these questions were not part of the current study. Only information from the self-efficacy instrument, knowledge application instrument, and communication instrument were utilized for this study and did not contribute to student grades.One of the interactive cases was written by the adolescent medicine faculty member responsible for the computer and interactive lectures in conjunction with oversight from the pediatric clerkship director. This case was adapted from a case previously written for educational experiences with pediatric residents.^21^ After their OSCE, students again completed the individual self-efficacy assessment.In addition to student completion of the OSCE examination, the standardized patient posing as an adolescent patient for the OSCE case completed a communication tool for each medical student to assess how well students communicated with the patient in the simulated learning case. Adolescent medicine faculty watched video of each medical student-standardized patient encounter to assess knowledge utilizing a knowledge usage assessment tool. Three students were missing video footage and their data was excluded from knowledge usage analysis.Therefore, the self-efficacy instrument was completed by students at the beginning of the rotation and at the conclusion of their OSCE. The knowledge application assessment was completed by faculty monitoring the OSCE or watching the video of the OSCE after the test, and the communication instrument was completed by the standardized patients at the conclusion of each student interaction during the OSCE.Investigators reviewed data for accuracy; any discrepancies were reviewed utilizing the paper versions of the assessment instruments and recorded videos of each subject. This research was approved by the institutional review board of the University of Arkansas for Medical Sciences in Little Rock, Arkansas.

### Participants and setting

Third-year medical students from the local college of medicine (n=64) were included in this study as they completed their two-month Pediatrics clerkship. Students completed their pediatric clerkship between March 2011-October 2011 (one group during this time period was excluded as self-efficacy forms were erroneously not completed on the day of the OSCE). Each two-month block included approximately 20 students. The rotation included one-month of inpatient pediatric care and one month of outpatient/nursery care. The outpatient month included one week of nursery, one week of general pediatric clinic, and two weeks of subspecialty clinics, including adolescent medicine. Students worked with patients in adolescent medicine clinic for one to three 4-hour sessions during their clerkship.

### Data collection procedures

#### Assessment instruments

A self-efficacy instrument, working with adolescent patients: Medical Student Self-Efficacy Questionnaire, was developed based upon existing, validated and reliable instruments that addressed adolescent health risk behaviors.^12^^,^^21^ In these existing tools, medical professionals were assessed on screening, risk assessment, counseling, referrals and follow-up of adolescent patients. The self-efficacy instrument contained seven items related to interviewing and communicating with adolescents regarding confidentiality, STI as well as risk assessment (4-item Likert scale from not at all confident to very confident). Students were given the instrument on day one of their pediatric clerkship during orientation. Students completed the assessment for a second time at the conclusion of their OSCE examination on the last day of their two-month clerkship in pediatrics.A communication instrument, the Patient-Physician Interaction Evaluation, was utilized to assess student communication skills with standardized patients. This instrument was adapted from an established validated and reliable tool written by the American Board of Internal Medicine (ABIM) Continuous Professional Development Program and was utilized for all simulations within the simulation center at the university.^22^ This instrument contained twelve items rating satisfaction with the student learner (5-item Likert scale ranging from poor to excellent). Standardized patients completed the instrument immediately after their interaction with the medical student during the OSCE.Finally, a knowledge application tool, the Adolescent Medicine OSCE Student Checklist, was utilized to assess student knowledge usage as they interacted with the standardized patients. A previous version of this instrument (validated and reliable) was created by study investigators^21^ and was adapted from an existing tool that focused on fourteen lifestyle areas.^23^ The tool was modified to apply to medical students and consisted of 11-items relating to care with adolescents especially with gynecologic, STI and contraception issues. Study investigators utilized video monitoring to watch the student interactions with SPs and were able to rate students (3-item scale: not addressed, not fully addressed, fully addressed) for each item. Videos were reviewed after the completion of the OSCE as to not interfere with the student testing process.

#### Educational sessions and learning activities

Two learning activities were developed by study investigators to assist student learners in their educational process working with adolescent patients. The first was a 60-minute PowerPoint lecture with audio narrative provided by the lead investigator. The lecture contained educational information on adolescent medicine topics including growth and development, eating disorders, contraception, STI, sports medicine and confidentiality.The study investigators also created a 60-minute case-based learning activity as an adjunct for the PowerPoint lecture. This case-based activity was interactive and facilitated by the lead investigator. Brief patient adolescent patient scenarios were presented on a PowerPoint slide to each group of approximately twenty learners for each two-month block in the pediatric rotation. Open-ended questions regarding medical concerns, history, examination, laboratory and testing, treatment, and plan were facilitated by the session moderator. Each case interaction had an approximate twenty-minute time allotment. Five scenarios were created to allow for variations in interaction of learning groups. The cases addressed sexual activity and STI, normal growth and development, eating disorders, amenorrhea, and contraception. Two groups of medical student learners received both the PowerPoint lecture and the interactive case-based activity. A third group received the PowerPoint lecture only.

#### Simulated Patient Module for OSCE

An adolescent case was developed for the end-of-rotation OSCE completed by each two-month block of students at the conclusion of their pediatric rotation. The case was conducted at the hospital simulated learning center as part of the end of rotation OSCE. Therefore, students were aware they were participating in an educational activity with SPs portraying patients, including the adolescent medicine case with the SP portraying an adolescent. Each SP was placed in the mock exam room with their chart available outside the door for review at the beginning of the module. Students were provided paper and a clipboard in order to take notes during the encounter. There was a 10-minute time limit for the case.

The case was a face-to-face encounter centered on gynecology issues in adolescent medicine. The standardized patient played the role of a 16-year old female patient visiting the clinic for the chief complaint of vaginal discharge. She also described symptoms of abdominal pain, nausea and poor appetite. The case was completed by each student during the OSCE, although the completion order was based upon the starting station for each student during the exam.

#### Standardized patients

The simulated learning center recruited SPs for the adolescent medicine case. Each SP was already utilized for other standardized patient exercises with the simulated learning center. The ‘teen’ SPs were either adolescents or individuals trained to portray adolescents. Over the course of the study, three SPs portrayed the teen in the OSCE case. A written scenario was provided to each potential SP for review, and study investigators were available to answer any questions or make terminology clarifications regarding the case. They were also provided with medical, social, family history in order to allow for development of a complete background.

### Analysis

The study assessed frequencies by group for self-efficacy at the beginning and end of the rotation, communication skills as determined by SPs, and knowledge usage measured by adolescent medicine faculty. Descriptive statistics were used to present this data. Additionally, comparisons were made between groups that received the recorded PowerPoint lecture plus interactive case based activity and the group that received only the recorded PowerPoint lecture. These comparisons were made for self-efficacy at both the beginning and end of the rotation, communication and knowledge utilizing a two sample t-test. (SPSS, 19.0)

## Results

### Self-efficacy

Evaluating medical student self-efficacy at the beginning of the two-month rotation (Table 1), students who had the case-based activity and the PowerPoint Lecture reported their highest levels of ‘no confidence’ were in assessing risk status for adolescents (16/42, 38%). The lowest ‘no confidence’ levels were for interviewing adolescents (1/42, 2%). Students receiving both lectures were rarely very confident for self-efficacy variables, and the highest level was only 7% (3/42) for asking personal questions and explaining confidentiality to adolescents. Considering both confident and very confident responses at the beginning of the rotation, students had only one variable over 50%-explaining confidentiality to adolescents (9/42, 52%).

**Table 1:** Self-efficacy frequencies for two-lecture group (n=42)

Adolescent self-efficacy variables		Not confident		Somewhat confident		Confident		Very confident	
		PRE	END	PRE	END	PRE	END	PRE	END
Interviewing adolescents	%	2	0	62	9	31	41	5	50
	Ratio	1/42	0/42	26/42	4/42	13/42	17/42	2/42	21/42
Asking personal questions	%	12	0	57	7	24	50	7	43
	Ratio	5/42	0/42	24/42	3/42	10/42	21/42	3/42	18/42
Assessing risk status	%	38	0	55	10	5	51	2	39
	Ratio	16/42	0/41	23/42	4/41	2/42	21/41	1/42	16/41
STI risk counseling	%	19	0	60	12	17	46	5	42
	Ratio	8/42	0/41	25/42	5/41	7/42	19/41	2/42	17/41
Pregnancy counseling	%	17	0	62	10	19	38	2	51
	Ratio	7/42	0/41	26/42	4/41	8/42	16/41	1/42	21/41
Contraception counseling	%	17	0	62	12	19	43	2	45
	Ratio	7/42	0/40	26/42	5/40	8/42	17/40	1/42	18/40
Confidentiality to adolescents	%	7	0	41	5	45	34	7	61
	Ratio	3/42	0/41	17/42	2/41	19/42	14/41	3/42	25/41
Discussing testing/treatment of STI	%	21	0	64	15	12	49	2	37
	Ratio	9/42	0/41	27/42	6/41	5/42	20/41	1/42	15/41
Counseling on STI prevention	%	14	0	60	10	21	39	5	51
	Ratio	6/42	0/41	25/42	4/41	9/42	16/41	2/42	21/41

At the end of the rotation, students receiving both lectures had 0% responses with a ‘not confident’ level. Although only very confident >50% for pregnancy counseling (21/41, 51%), counseling on STI prevention (21/41, 51%), and confidentiality with adolescents (25/41, 61%), students were either confident or very confident >85% for all variables at the end of the rotation.Students who received only the PowerPoint lecture (Table 2) reported the highest level of no confidence in regard to assessing risk status (3/22, 14%). Over one-fourth of students in this group stated they were ‘very confident’ explaining confidentiality to adolescents (6/22, 27%), and 50% were either confident or very confident in this area. Nearly 90% of students were either confident or very confident discussing STI treatment (19/22, 87%). Students had no other levels over 50%.

**Table 2:** Self-efficacy frequencies for one-lecture group (n=22)

Adolescent self-efficacy variables		Not confident		Somewhat confident		Confident		Very confident	
		PRE	END	PRE	END	PRE	END	PRE	END
Interviewing adolescents	%	9	0	50	23	41	50	0	27
	Ratio	2/22	0/22	11/22	5/22	9/22	11/22	0/22	6/22
Asking personal questions	%	9	0	64	27	23	41	4	32
	Ratio	2/22	0/22	14/22	6/22	5/22	9/22	1/22	7/22
Assessing risk status	%	14	0	54	27	32	50	0	23
	Ratio	3/22	0/22	12/22	6/22	7/22	11/22	0/22	5/22
STI risk counseling	%	4	0	82	32	13.5	41	0	27
	Ratio	1/22	0/22	18/22	7/22	3/22	9/22	0/22	6/22
Pregnancy counseling	%	4	0	68	23	23	41	4	36
	Ratio	1/22	0/22	15/22	5/22	5/22	9/22	1/22	8/22
Contraception counseling	%	4	0	64	27	27	50	4	23
	Ratio	1/22	0/22	14/22	6/22	6/22	11/22	1/22	5/22
Confidentiality to adolescents	%	0	0	50	23	23	46	27	32
	Ratio	0/22	0/22	11/22	5/22	5/22	10/22	6/22	7/22
Discussing testing/treatment of STI	%	0	0	14	32	64	45	23	23
	Ratio	0/22	0/22	3/22	7/22	14/22	10/22	5/22	5/22
Counseling on STI prevention	%	0	0	59	23	32	45	9	32
	Ratio	0/22	0/22	13/22	5/22	7/22	10/22	2/22	7/22

At the end of the rotation, students had no ‘not confident’ responses for any variable. There were also no students who were very confident for any response >50%; pregnancy counseling had the highest response at 36% (8/22). The combination of confident and very confident responses for all variables was also lower for this group with all variables >65% compared to the 85% for students with both lectures.

### Knowledge application

For the students receiving both lecture types, no students (0/42, 0%) forgot to address physical symptoms, sexual activity, and contraception as seen in Table 3. Among this same group, 36% did not address pregnancy (14/39). Students asked about physical symptoms 100% of occasions, although they only asked about pregnancy and sexual activity 23% (9/39) and dating 21% (8/39).

**Table 3:** Knowledge application levels for two-lecture (n=39) and one-lecture groups (n=22)

Knowledge variable		Not addressed		Not fully addressed		Fully addressed	
		Two-Lecture Group	One-Lecture Group	Two-Lecture Group	One-Lecture Group	Two-Lecture Group	One-Lecture Group
Menstrual History	%	13	18	46	46	62	36
	Ratio	5/39	4/22	10/39	10/22	24/39	8/22
Physical Symptoms	%	0	0	0	4	100	96
	Ratio	0/39	0/22	0/39	1/22	39/39	21/22
Vaginal Symptoms	%	28	36	10	0	62	64
	Ratio	11/39	8/22	4/39	0/22	24/39	14/22
Sexual Activity	%	0	0	71	68	23	32
	Ratio	0/39	0/22	30/39	15/22	9/39	7/22
Dating	%	8	9	42	82	21	9
	Ratio	3/39	2/22	28/39	18/22	8/39	2/22
Contraception	%	0	0	13	9	87	91
	Ratio	0/39	0/22	5/39	2/22	34/39	20/22
Pregnancy	%	36	14	41	59	23	27
	Ratio	14/39	3/22	16/39	13/22	9/39	6/22
STI Testing	%	8	4	51	73	41	23
	Ratio	3/39	1/22	20/39	16/22	16/39	5/22
STI Risks	%	18	46	56	36	26	18
	Ratio	7/39	10/22	22/39	8/22	10/39	4/22
STI Prevention	%	15	9	15	41	69	50
	Ratio	6/39	2/22	6/39	9/22	27/39	11/22
Confidentiality	%	3	18	62	55	36	27
	Ratio	1/39	4/22	24/39	12/22	14/39	6/22

Students receiving only the PowerPoint lecture did not address STI risks for 67% (10/15) and vaginal symptoms for 40% (6/15) of encounters. Sexual activity, dating, contraception, STI testing, and STI prevention were addressed to some extent by these students. Students fully addressed contraception and STI testing (15/22, 68%). Unfortunately, 0% of the students fully addressed dating, and only 13% (2/15) fully addressed STI risk factors.

### Communication skill

Students receiving both the PowerPoint lecture and the interactive case-based lecture were never rated as poor by the standardized patients, although at least one student was rated as ‘fair’ for 8 of 10 variables (Table 4). Ten percent (4/42) of students were rated as fair for a warm attitude. The highest rating for students with both lectures was for eye contact, where SPs rated students as ‘excellent’ in 29% (12/42) of encounters. Combining good plus excellent ratings, students were rated highly for listening (34/42, 81%); however, only 45% (19/42) were deemed to have given a proper introduction.

**Table 4:** Communication skill for two-lecture (n=42) and one-lecture groups (n=22)

Communication Variable		Poor		Fair		Good		Very good		Excellent	
		2-Lecture	1-Lecture	2-Lecture	1-Lecture	2-Lecture	1-Lecture	2-Lecture	1-Lecture	2-Lecture	1-Lecture
Proper introduction	%	0	5	0	9	55	27	29	55	17	5
	Ratio	0/42	1/22	0/42	2/22	23/42	6/22	12/42	12/22	7/42	1/22
Confidence	%	0	0	5	5	38	23	43	55	14	18
	Ratio	0/42	0/22	2/42	1/22	16/42	5/22	18/42	12/22	6/42	4/22
Warm attitude	%	0	0	10	14	36	9	33	36	21	41
	Ratio	0/42	0/22	4/42	3/22	15/42	2/22	14/42	8/22	9/42	9/22
Respect	%	0	0	2	0	24	23	60	73	14	5
	Ratio	0/42	0/22	1/42	0/22	10/42	5/22	25/42	16/22	6/42	1/22
Nonjudgmental	%	0	0	5	0	26	23	57	77	12	0
	Ratio	0/42	0/22	2/42	0/22	11/42	5/22	24/42	17/22	5/42	0/22
Interest	%	0	0	7	5	43	41	31	46	19	9
	Ratio	0/42	0/22	3/42	1/22	18/42	9/22	13/42	10/22	8/42	2/22
Bored	%	0	0	2	0	36	14	48	77	14	9
	Ratio	0/42	0/22	1/42	0/22	15/42	3/22	20/42	17/22	6/42	2/22
Eye contact	%	0	0	2	0	21	32	48	41	29	6
	Ratio	0/42	0/22	1/42	0/22	9/42	7/22	20/42	9/22	12/42	6/22
Term explanation	%	0	0	5	0	38	41	45	50	12	9
	Ratio	0/42	0/22	2/42	0/22	16/42	9/22	19/42	11/22	5/42	2/22
Listened	%	0	0	0	0	19	36	64	50	17	14
	Ratio	0/42	0/22	0/42	0/22	8/42	8/22	27/42	11/22	7/42	3/22

For students receiving only the PowerPoint lecture, one student was rated ‘poor’ by an SP for giving a proper introduction. Students received a ‘fair’ rating for four communication variables: displaying a warm attitude (3/22, 14%), proper introduction (2/22, 9%), demonstrating confidence (1/22, 5%), and appearing interested (1/22, 5%). There were no excellent ratings for being nonjudgmental and 41% (9/22) students received an excellent rating for having a warm attitude. Combining very good and excellent ratings, students had the highest rating for not appearing bored (19/22, 86%) and the lowest rating for appearing interested (12/22, 55%).

**Table:** 

### **Comparisons between Medical Student Groups:** Self-Efficacy, Knowledge Application, and Communication Skill

### Self-efficacy

Comparing self-efficacy at the beginning of the pediatric clerkship for the medical students receiving the PowerPoint Lecture and the case-based lecture v. medical students receiving only the PowerPoint lecture, one variable showed a significant difference between the two groups (Table 5). Medical students that received only the PowerPoint lecture had higher self-efficacy levels in assessing adolescent risk than students receiving both lectures (t_(43)_=2.662, p=.01). Of marginal significance, medical students receiving only the PowerPoint lecture also had higher self-efficacy levels on counseling STI prevention (t_(46)_=1.828, p=.07).

**Table 5:** Comparison of self-efficacy differences between groups

Self-efficacy variable	Pre rotation		End rotation	
	t_(df)_	Sig	t_(df)_	Sig
Interviewing adolescents	-0.374_(41)_	0.71	-1.942_(40)_	0.059^**^
Asking personal questions	-0.184_(47)_	0.86	-1.618_(35)_	0.12
Assessing risk status	2.662_(43)_	0.01^*^	-1.840_(39)_	0.07^**^
STI risk counseling	0.133_(61)_	0.90	-1.705_(38)_	0.096^**^
Pregnancy counseling	1.182_(45)_	0.24	-1.424_(38)_	0.16
Contraception counseling	1.426_(45)_	0.16	-1.958_(42)_	0.057^**^
Confidentiality with adolescents	1.143_(37)_	0.26	-2.543_(35)_	0.02^*^
Discussing testing/treatment STI	0.838_(46)_	0.41	-1.619_(40)_	0.12
Counseling on STI prevention	1.828_(46)_	0 .07^**^	-1.694_(39)_	0.098^**^

^*^p<0.05, ^**^p<0.1

Students receiving both lectures had significantly higher self-efficacy levels discussing confidentiality with adolescents (t_(35)_=-2.543, p=.02) at the end of the pediatric clerkship. Of marginal significance, students receiving both lectures also had higher self-efficacy levels for interviewing adolescents (t_(40)_=-1.942,p=.059), assessing risk factors (t_(39)_=-1.840, p=.07), counseling on STI risk (t_(38)_=-1.705, p=.096), counseling on contraception (t_(42)_=-1.95, p=.057), and counseling on STI prevention (t_(39)_=-1.694, p=.098).

### Knowledge application

Of marginal significance, students receiving two lectures had higher knowledge application levels addressing STI risks with SPs during their OSCE at the end of the pediatric clerkship than students receiving only the PowerPoint presentation (t_(39)_=-1.792, p=.08). There were no significant differences between knowledge application levels in the two groups for any other items.

### Communication skill

No significant differences were elicited between the two groups in communication skill as measured by SPs during the OSCE.

## Discussion

Our data highlight that medical learners are often unaffected by the medium in which knowledge is presented to them in an educational setting. Overall, the findings suggest that medical students are adaptable to the information they are taught and presented, and subsequently are able to perform in a manner that successfully exhibits both knowledge application and communication skill at comparable levels.Communication skill levels were equal for students receiving only the PowerPoint lecture and students receiving both the PowerPoint and the interactive case-based activity. Although no significant differences were discovered between groups, the similarities are noteworthy. Neither group was rated as ‘excellent’ for any communication item >50% by SPs during the simulated examination. Combining very good and excellent responses by SPs, students from each group were rated <90% for all items.Knowledge application was similar for both lecture groups, although there was a trend toward better knowledge usage in the double lecture group for explaining STI risks. Such findings could be explained by detailed discussion of patient examples in the case-based interactive activity with focus on active learning rather than passive knowledge, as previously described by Jones et al.^24^ Similar findings were seen in previous work with case learning in comparison with problem based learning.^25^^,^^26^Both lecture groups displayed strengths in fully addressing physical symptoms and contraception in over 85% of SP encounters. Weakness was exhibited by both groups for multiple topics with five of ten knowledge variables addressed <10% for both groups. In the standardized OSCE case of an adolescent patient presenting with vaginal discharge, menstrual history, vaginal symptoms, pregnancy, and STI risks were often not discussed by the students.Self-efficacy exhibited the most contrast between the two groups. Although self-efficacy was significantly higher for the PowerPoint lecture group at the beginning of the rotation, no self-efficacy levels were higher for this group compared to the two lecture group at the end of the pediatric clerkship. The two-lecture group of medical students did have significantly higher self-efficacy explaining confidentiality to adolescents and marginally significant higher self-efficacy regarding interviewing, assessing risk, counseling on STI risk, prevention and contraception. Such differences suggest that an interactive case-based activity allows for examination of real-life scenarios that might not be available with only a PowerPoint lecture. Previous studies also exhibited that students with case study learning exhibited higher-order thinking skills and better test taking on higher difficulty test questions.^27^^, ^^28^Medical students in the two-lecture group were ‘very confident’ at the end of the clerkship >50% for four items (interviewing adolescents, pregnancy counseling, explaining confidentiality to adolescents, and counseling on STI prevention) whereas the one-lecture group’s highest ‘very confident’ answer percentage was 36% for pregnancy counseling. Examining the combination of ‘very confident’ plus ‘confident’ responses by group, the two-lecture group had >85% self-efficacy for all items, but the one-lecture group was only >67% for all items.Such findings suggest that while knowledge application and communication skill may be the same for students receiving lectures in different mediums or a different quantity of lectures, self-efficacy actually differs. The learner’s confidence in personal ability to perform tasks appears to be higher when participating in an interactive learning environment. Additionally, according to Social Cognitive theory, individuals are more likely to participate in future events in areas where they feel comfortable and confident. Therefore, students with higher self-efficacy levels may actually seek to ask questions of future patients on sensitive medical topics that affect their general well-being and health risk in the future. Such findings resonate with Sutyak et al’s (1993) work which exhibited that students exposed to unstructured case learning felt more at ease and enjoyed learning in situations similar to reality, allowing them to feel more like ‘real doctors’.^29^Our study emphasizes that confidence can increase through practice and interaction, and though the short-term outcomes may be similar, the impact on subsequent and future physician-patient interactions in real world situations may be considerable.

### Limitations

Limitations of our study should be considered. We had a relatively small study population which may have affected overall analysis as well as generalizability to all populations. Learning of any method is dependent on the teacher, and there was only one instructor for this study. Case-based learning methods can also be affected by personalities of the learners, especially with more dominating personalities.^30^ Additionally, our subjects interacted with SPs on one occasion. While studies have shown that SPs provide quality assessments of first impressions with practitioners, their ability to assess future interactions is unknown.^31^ By nature of design, the medical student OSCE assesses knowledge application and skill at the end of the pediatric rotation, limiting multiple interactions with SPs.

## Conclusions

Our findings suggest that differing learning formats may have an effect on a medical learner’s self-efficacy leading to future change in interactions with patients. Case-focused learning allows medical students the opportunity to experience ‘real cases’ with interactive patient experiences and outcomes compared to pre-prepared traditional lectures. As there are no required rotations for medical students in Adolescent Medicine, the importance of conveying information that is absorbed in a short amount of time is paramount, and case-focused learning may be the best format for this process.Although communication and knowledge usage appear to be unaffected by learning format, self-efficacy showed a change with usage of an interactive case-based format in addition to a more standard PowerPoint lecture format. In comparison to knowledge and communication, self-efficacy is rarely utilized as a marker for future success for medical providers, but may be an important element of the educational process. Medical educators may wish to study self-efficacy’s role in learning over the entire medical learning process to assess potential effects on education and career trajectories.

### Acknowledgements

All the employees at Arkansas Children’s Hospital PULSE Center for allowing us to perform our study.

### Conflict of Interest

The authors declare that they have no conflict of interest.
